# LILBID laser dissociation curves: a mass spectrometry-based method for the quantitative assessment of dsDNA binding affinities

**DOI:** 10.1038/s41598-020-76867-9

**Published:** 2020-11-23

**Authors:** Phoebe Young, Genia Hense, Carina Immer, Jens Wöhnert, Nina Morgner

**Affiliations:** 1 Institute of Physical and Theoretical Chemistry, J.W. Goethe University, 60438 Frankfurt am Main, Hesse Germany; 2 Institute for Molecular Biosciences, J.W. Goethe University, 60438 Frankfurt am Main, Hesse Germany; 3 Center for Biomolecular Magnetic Resonance (BMRZ), J.W. Goethe University, 60438 Frankfurt am Main, Hesse Germany

**Keywords:** Biochemistry, Mass spectrometry, Chemistry, Physical chemistry

## Abstract

One current goal in native mass spectrometry is the assignment of binding affinities to noncovalent complexes. Here we introduce a novel implementation of the existing laser-induced liquid bead ion desorption (LILBID) mass spectrometry method: this new method, LILBID laser dissociation curves, assesses binding strengths quantitatively. In all LILBID applications, aqueous sample droplets are irradiated by 3 µm laser pulses. Variation of the laser energy transferred to the droplet during desorption affects the degree of complex dissociation. In LILBID laser dissociation curves, laser energy transfer is purposely varied, and a binding affinity is calculated from the resulting complex dissociation. A series of dsDNAs with different binding affinities was assessed using LILBID laser dissociation curves. The binding affinity results from the LILBID laser dissociation curves strongly correlated with the melting temperatures from UV melting curves and with dissociation constants from isothermal titration calorimetry, standard solution phase methods. LILBID laser dissociation curve data also showed good reproducibility and successfully predicted the melting temperatures and dissociation constants of three DNA sequences. LILBID laser dissociation curves are a promising native mass spectrometry binding affinity method, with reduced time and sample consumption compared to melting curves or titrations.

## Introduction

Native mass spectrometry (MS) was introduced in the 1990s as a method to study intact noncovalent biomolecular complexes^1,2^, focusing primarily on their stoichiometry. However, to understand the function of such complexes, it is important not only to know their composition but also to characterize the binding affinities of their components. In recent years, several studies have been published seeking to extend native mass spectrometry to the determination of binding affinities in the gas^3–5^ and solution phases^5–7^, using the most widespread native mass spectrometry method, nano-electrospray ionisation mass spectrometry (nESI-MS). A newer native mass spectrometry method, laser-induced liquid bead ion desorption mass spectrometry (LILBID-MS), was initially introduced by Kleinekofort et al. as laser-induced liquid beam ion desorption mass spectrometry in 1996^8,9^ and re-introduced as laser-induced liquid bead ion desorption mass spectrometry in 2006^10^. In the LILBID ion source, microdroplets of an aqueous sample are produced by an on-demand droplet generator at a rate of 10 Hz. Each droplet is transferred into the ion source and irradiated by a pulse of infrared laser light (6 ns pulses at a rate of 10 Hz, wavelength: 3 µm). Laser light is absorbed by the first few nm of each aqueous droplet, leading to an explosive expansion moving as a shock wave through the volume of the droplet^10,11^. During this process, some of the sample molecules are desorbed from their water matrix and brought into the gas phase for mass analysis. Since its introduction, LILBID-MS has proven to be a suitable complement to the more widespread nESI-MS as a native mass spectrometry method^12^ and has been successfully used to determine the stoichiometries of noncovalent biomolecular complexes^13–17^. In this study, we developed a LILBID-based method for assessing the binding affinities of such complexes.

Existing strategies for measuring binding affinities in solution often centre on titration or melting studies^7,18–25^. Titrations are based on the concept that changing the concentration of one component in a binding reaction results in observable shifts in the equilibrium between the complex and the binding partners. In melting curve experiments, shifts in equilibrium are achieved by increasing the temperature (kinetic energy) of the sample. In each of these analyses, the resulting changes in the equilibrium are plotted against the independent variable. Binding affinity is assigned from the shape or position of the resulting curve. These existing methods, including those based on native mass spectrometry techniques, have different advantages and drawbacks^7,21–25^. Given the existing strategy of using melting curves, the intuitive approach was to use LILBID-based melting curves. Therefore, we initially took this approach on this project: aqueous samples were heated and equilibrated within the droplet generator prior to LILBID analysis. However, collecting melting curves necessitates the scanning of a large temperature range. Since the heatable droplet generator has no cooling function and does not yield stable droplets above 60 °C, full LILBID melting curves were only accessible for samples in a small range of melting temperatures (T_m_s) (Supplementary Fig. S1). These T_m_s were the first quantitative binding affinities assessed with LILBID. However, given their limited range, the heatable droplet generators did not seem to be the ideal means to transfer the necessary energy to the samples. These results encouraged us to follow an altered approach, which is the focus of this paper.

We now introduce the LILBID laser dissociation curve method, a method that centers around the IR laser used in LILBID. In the LILBID ion source, irradiating aqueous sample droplets with IR laser light results in energy transfer to the droplet and to the analyte molecules. Two effects of this laser energy transfer have been observed. It leads to desorption of solvated ions from the droplet, releasing them into the gas phase for analysis. Depending on the amount of laser energy transferred to the droplets, analytes may also acquire enough energy for covalent bonds to break (fragment) or for noncovalent bonds to dissociate. Since fragmentation of covalent bonds is rarely observed in LILBID, we speak primarily of the dissociation of noncovalent bonds. The mechanism of laser energy transfer, which leads to desorption and laser-induced dissociation, are the subject of on-going research^26–30^. Nevertheless, past qualitative studies have shown that different amounts of laser energy transfer lead to differences in complex dissociation^12,13,31,32^. To dissociate noncovalent interactions with stronger binding affinities more laser energy transfer is required^12^. By increasing laser energy transfer, the amount of dissociation can be increased in a controlled way. In a LILBID laser dissociation curve, this relationship between laser energy transfer and dissociation is put to use; laser energy transfer is deliberately varied and the resulting dissociation plotted as a function of this energy transfer.

One rather challenging requirement for the creation of LILBID laser dissociation curves is the quantification of the amount of energy transferred to the sample. Calculating the amount of laser energy transfer to each droplet and to the analyte molecules in each droplet is not possible since the exact mechanism of LILBID is not known. Further, several unavoidable factors affect laser energy transfer: fluctuations in the laser pulse or power and in droplet position relative to the laser focus. In this paper, we make use of the shape of the droplet explosion as a robust observable that is well correlated with laser energy transfer. Rather than calculating laser energy transfer for each droplet, we use the observed droplet explosion width as a proxy for laser energy transfer in the LILBID laser desorption process. In this way, all factors that affect laser energy transfer, including experimentally unavoidable fluctuations, are taken into account. Using this approach to assess laser energy transfer, LILBID laser dissociation curves plot dissociation as a function of laser energy transfer to find binding affinities.

We present the binding affinities calculated for dsDNA from the LILBID laser dissociation curves and compare the results to those from two solution phase methods: UV melting studies and isothermal titration calorimetry (ITC). We demonstrate that the binding of dsDNA samples assessed with the new method strongly correlates with results from the two solution state methods and use the LILBID laser dissociation curve method to predict the T_m_ and dissociation constant (K_d_) values for three dsDNA samples.

## Results

For a first test of the LILBID laser dissociation curve method we chose a set of DNA double strands for two reasons. Firstly, DNA sequences can be easily designed to cover a range of different binding affinities, and secondly, they allow the use of not only ITC but also UV melting curves as established binding affinity methods for comparison with our method. UV melting curves represent a standard method of measuring the binding affinity of oligonucleotides in the solution state, while ITC provides an additional, room temperature method for comparison. Twelve double-stranded DNA oligonucleotide samples were prepared (Table 1), and their binding strengths were evaluated with these three different methods: UV melting curves, ITC, and LILBID laser dissociation curves. Results from the LILBID laser dissociation curves were correlated with affinities calculated from UV melting curves and from ITC.

**Table 1:** Sequences and masses of DNA oligonucleotides used.

Oligonucleotide	Sequence	Mass [Da]
9_Amer	CAT AAT CAA	2700
10_Amer	TAC TAA AAA C	3004
11_Amer	CAT AAT CAA CT	3284
15_Amer	TAC TAA AAA CAT AAT	4552
15b_Amer	CTC AAA AAA ACT ACA	4522
20_Amer	TCA ACT CAA AAA AAC TAC AA	6055
9_Bmer	CCG TAA TCT	2674
10_Bmer	CGT AAT CTC A	2987
12_Bmer	CCG TAA TCT CAC	3565
8_Cmer	GAA GAG CC	2444
10_Cmer	AGA AGA GCC G	3086
12_Cmer	TGA GAA GAG CCG	3720
35_Amer	ATT GTA GTT TTT TTG AGT TGA TTA TGT TTT TAG TA	10,823
35_Bmer	TTT TGT GAG ATT ACG GAA CCT TTT TTT TTT TTT GT	10,735
35_Cmer	TTT TAT CGG CTC TTC TCA TTT TAT TTT ATT TTG TT	10,621

### UV melting curves and ITC yielded T_m_s and K_d_s as comparison points for LILBID laser dissociation curves

DNA melting curves were obtained with UV spectroscopy to determine the melting temperatures of 12 different dsDNA samples as a measure of their solution phase binding affinity. The UV curves and the melting temperatures from these curves show that the set of chosen dsDNA samples covers a large range of binding affinities (Table 2, Supplementary Figs. S2 and S3). As expected, both longer dsDNA sequences and higher GC content (e.g. compare 15_Amer and 15b_Amer measurements) generally correlate with higher melting temperatures and a corresponding shift in the melting curves to higher temperatures. ITC curves were also collected for ten of these dsDNA samples; the resulting dissociation constants were well correlated with the melting temperatures (Table 2, Supplementary Figs. S4 and S5). The binding affinities of the 12_Cmer + 35_Cmer sample and the 20_Amer + 35_Amer sample were not measured with ITC as they are beyond the range of the ITC.

**Table 2:** For each DNA double strand the percent GC content, theoretical and experimental melting temperatures, dissociation constants and % ssDNA observed with LILBID at 1.1 mm droplet explosion width are shown.

Shorter ssDNA	Scaffold ssDNA	% GC	Theor. T_m_ [°C]	UV T_m_ [°C]	ITC K_d_ [nM]	LILBID % ssDNA at 1.1 mm
9_Amer	35_Amer	22.2	22.4	22.7 ± 0.9 (2)	8500 ± 700 (3)	77 ± 3
10_Amer	35_Amer	20.0	26.7	26.0 ± 1.4 (2)	3900 ± 500 (3)	79 ± 2
11_Amer	35_Amer	27.3	34.3	34.6 ± 1.2 (2)	370 ± 50 (5)	65 ± 5
15_Amer	35_Amer	13.3	41.6	40.0 ± 1.4 (4)	31.2 ± 0.8 (3)	47 ± 4
15b_Amer	35_Amer	26.7	47.1	47.5 ± 0.4 (4)	3.6 ± 0.5 (3)	36 ± 3
20_Amer	35_Amer	25.0	56.2	55.8 ± 1.3 (2)	*	8 ± 3
9_Bmer	35_Bmer	44.4	31.4	33.3 ± 0.1 (2)	2120 ± 60 (3)	68.5 ± 0.9
10_Bmer	35_Bmer	40.0	35.1	35.3 ± 1.8 (2)	353 ± 7 (3)	70.6 ± 3
12_Bmer	35_Bmer	50	45.2	49.1 ± 1.3 (2)	20.1 ± 1.8 (3)	34.6 ± 1.4
8_Cmer	35_Cmer	62.5	30.7	34 ± 2 (4)	418 ± 16 (4)	61.7 ± 1.2
10_Cmer	35_Cmer	60.0	44.4	44 ± 2 (3)	29 ± 7 (3)	46 ± 3
12_Cmer	35_Cmer	58.3	51.4	52.6 ± 0.2 (2)	*	40.4 ± 1.9

Percent GC is shown for only the double-stranded section of each sample. Theoretical T_m_s were calculated for 10 µM dsDNA, 0.5 mM MgCl_2_ using https://eu.idtdna.com/calc/analyzer. UV T_m_s and ITC K_d_s are given as mean ± s.e.m., and the number of replicate UV or ITC curves is given in parentheses. Percent ssDNA at 1.1 mm represent the mean from five LILBID dissociation curves ± s.e.m.

*Analysis was not run.

### Explosion width as a measure of laser energy transfer

In LILBID-MS the amount of laser light irradiating the droplet can be controlled. This influences the amount of energy transferred to the sample and in turn affects both absolute peak intensity and the amount of dissociation observed in the resulting spectra. We postulated that this tunable energy transfer could be used to produce dissociation curves analogous to melting curves; instead of heating a sample to higher temperatures to cause dissociation, more energy could be transferred into the system via laser tuning, resulting in more dissociation. Complexes with higher binding affinities would require more laser energy transfer to reach a given amount of dissociation.

However, ensuring the transfer of an exact and predefined amount of laser energy transfer to a droplet is not trivial. Several factors affect the amount of laser energy transferred, including the power of the laser pulse, the position of the laser focus and the exact position of the droplet in the ion source at the time of the laser pulse, which influences the overlap between laser and droplet. The relationship between these factors and the amount of laser energy transferred to the droplet is not known. Thus, calculating laser energy transfer from experimental parameters is not possible. Further, unavoidable fluctuations in some parameters like droplet position complicate matters, making exact amounts of laser energy transfer difficult to reproduce. Rather than focusing on these experimental parameters, we took another approach to quantitatively vary and track laser energy transfer. We had observed that increased laser energy transfer led to more intense droplet explosions as well as increases in free gas phase ions and in complex dissociation. Thus, we investigated the shape of the droplet explosion as a potential measure of laser energy transfer.

**Figure 1:**
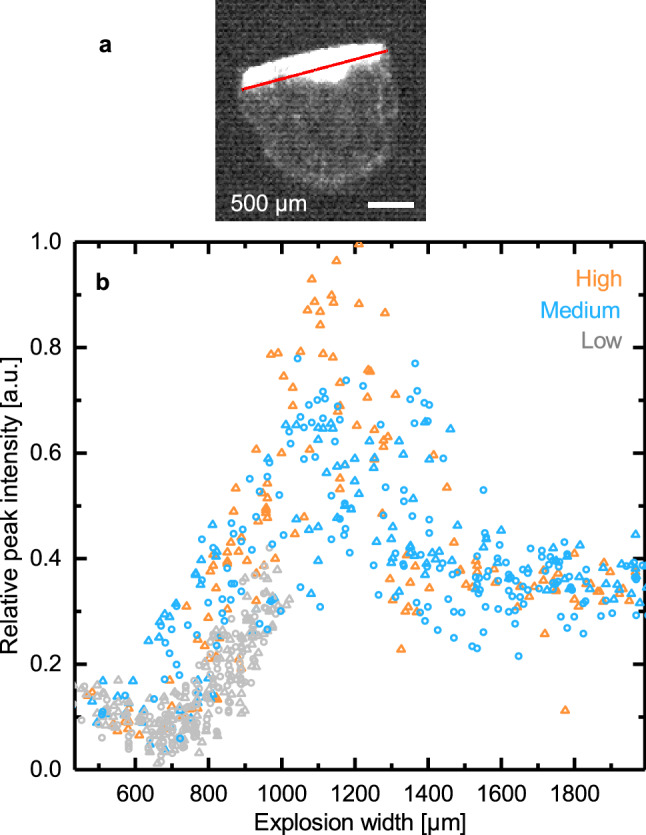
Peak area versus explosion width. (**a**) Explosion width of a droplet. Image taken 5 µs after IR pulse. Image contrast was enhanced for better visualization. (**b**) Peak intensity vs. explosion width for 35_Amer ssDNA. Different explosion widths were achieved by varying laser power and either adjusting droplet position relative to the desorbing IR laser beam within the flight axis (triangle) or adjusting droplet position relative to the laser beam perpendicular to the flight axis (open circle). This figure was prepared in OriginPro 2017 (https://www.originlab.com/). The image in (**a**) was prepared using Fiji^33^.

Because laser energy transfer affects both peak intensity and dissociation, we decided to investigate each parameter separately. To observe changes in peak intensity independent of dissociation, we used a 10 µM solution of the 35_Amer ssDNA in 0.5 mM MgHPO_4_. We tracked peak intensity while varying laser power and droplet position within the laser beam and imaged the droplets’ explosive expansions 5 µs after irradiation. To find an observable that would be a good measure of the transferred laser energy, we tried to correlate several parameters, such as the area, location, brightness, width and angle of the droplet explosion, with peak intensity in the resulting spectra. Peak area was strongly correlated with the width of the explosive expansion of the droplet 5 µs after irradiation (Fig. 1). Regardless of the parameters used to vary laser energy transfer (laser power or droplet position relative to the laser focus), the width of the droplet explosion was sufficient to predict peak area in the resulting spectrum, demonstrating that explosion width is a robust predictor of laser energy transfer (Fig. 1B). Conversely, sample and buffer concentration were not observed to affect explosion width (Supplementary Figs. S6 and S7).

The initial increase of peak area with increasing explosion width is in keeping with the thought that increasing laser energy, observed as wider explosions, should lead to more effective desorption of the sample from the droplet. The decrease in signal intensity above ~ 1200 µm points to an additional phenomenon likely competing with the increase in desorption (Fig. 1). One explanation is that this is an effect caused by our Wiley-McLaren type ion accelerator^34^: ions may have higher velocities if they originate from high energy droplet explosions (droplets with greater explosion widths). In the delay time before the voltages are applied to the ion optics to accelerate the ions towards the detector, those ions that have high kinetic energy and are also moving away from the ToF detector may fail to be successfully re-directed toward the detector (Supplementary Fig. S8). To avoid the complication of this secondary effect during data interpretation we will consider data taken from droplet explosions only in the range below ~ 1200 µm.

### Establishment of LILBID laser dissociation curves as a method of assessing DNA binding affinities

**Figure 2:**
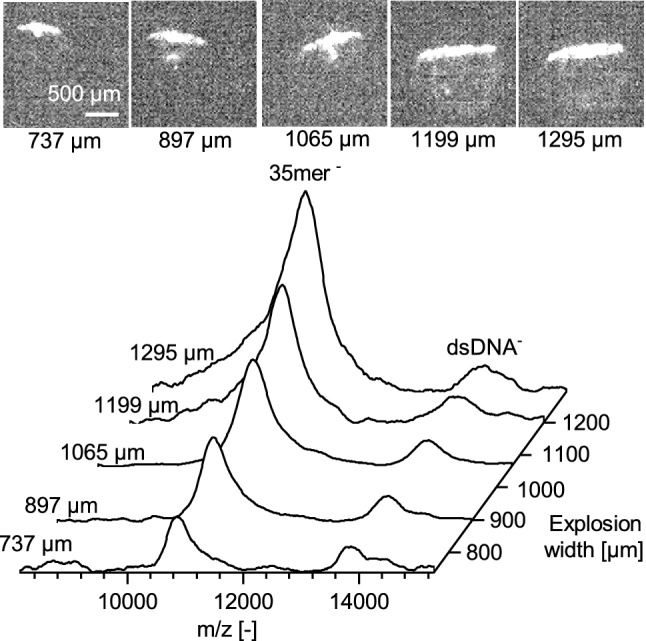
LILBID spectra of five droplets with different explosion widths. Top: Images of the corresponding droplets taken 5 µs after IR irradiation. Image contrast was enhanced for better visualization. Bottom: Spectra showing increasing dissociation of the 10_Amer + 35_Amer sample with increasing explosion width. Spectra are normalized to the peak height of the dsDNA^-^ peak. For full spectra, see Supplementary Fig. S9. This figure was prepared in OriginPro 2017 (https://www.originlab.com/). The images in the figure were prepared using Fiji^33^.

Explosion images and the corresponding spectra from five representative droplets from the 10_Amer + 35_Amer sample show that dissociation of the dsDNA increased with increasing explosion width in the range up to about 1200 µm (Fig. 2, Supplementary Fig. S9). Since explosion width can be taken as a proxy for the amount of laser energy transferred to the sample molecules, it is clear that with increased transfer of laser energy to the sample molecules, more dissociation is observed.

Knowing that laser energy transfer in LILBID can be varied in a controlled fashion and that this variation can be reliably monitored by tracking explosion width, the next step was to use laser-induced dissociation to develop an alternative to melting curve analyses. A set of DNA dissociation curves was collected with LILBID-MS, by varying the laser energy transferred rather than varying the temperature of the solution phase sample. LILBID spectra were collected while varying the droplet position within the laser beam and recording the images of the droplets’ explosions. The relative peak area for ssDNA was plotted against droplet explosion width to achieve a dissociation curve.

**Figure 3:**
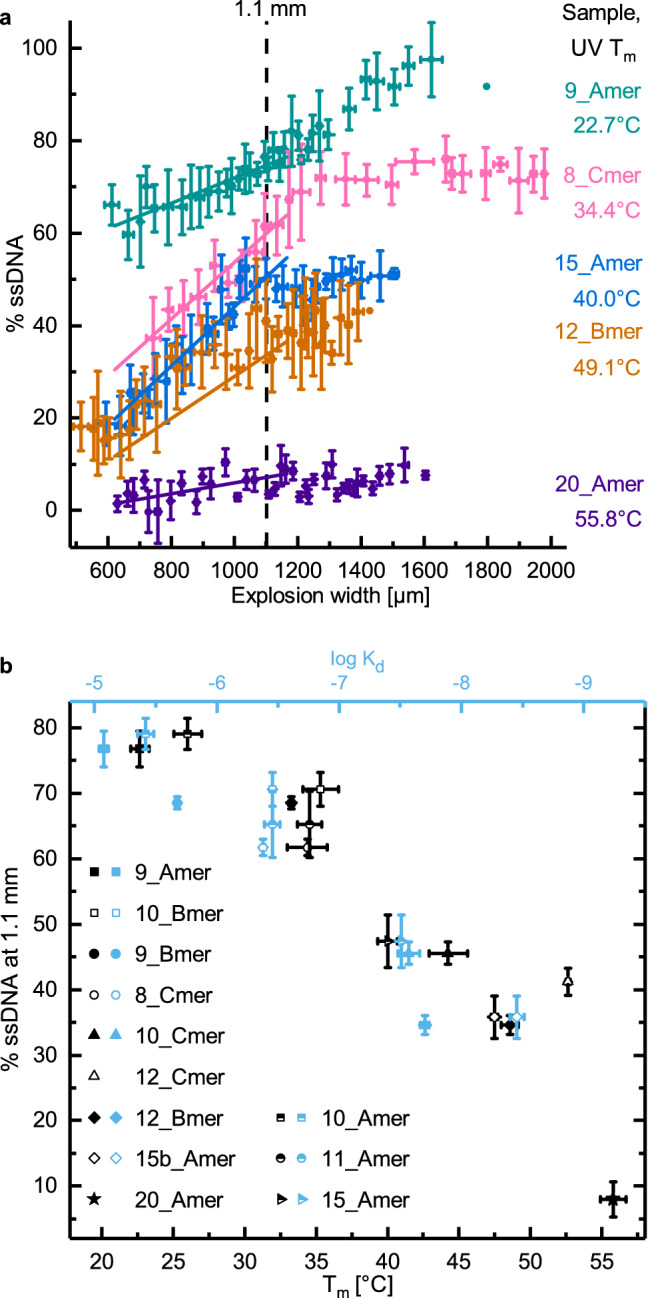
LILBID laser dissociation curves and comparison with UV and ITC results. (**a**) Laser dissociation curves with linear fits in the region of interest (620–1200 µm). This allows the interpolation of % ssDNA at the explosion width with the best spectral signal-to-noise (1.1 mm). The dotted line indicates this point; at this explosion width % ssDNA serves as a measure of binding affinity. For the sake of legibility, one laser dissociation curve is shown for each of five dsDNA samples. For information on calculations for this figure, see Supplementary Fig. S10. (**b**) Results from LILBID laser dissociation curves from 12 dsDNA samples. % ssDNA at 1.1 mm vs. solution state melting temperature (black) and vs. solution state log K_d_ (blue). All values are shown as mean ± s.e.m., calculated from five LILBID replicates, two to four melting curves and two to five ITC curves per sample. For information on calculations for this figure, see Supplementary Fig. S10. For UV melting curves and representative ITC curves, see Supplementary Figs. S2, S3, S4 and S5. This figure was prepared in OriginPro 2017 (https://www.originlab.com/).

LILBID laser dissociation curves were recorded for 12 dsDNA samples (six using the 35_Amer sequence, three with the 35_Bmer and three with the 35_Cmer). The trend of more dissociation occurring at increasing explosion widths up to about 1200 µm held for all dsDNA samples (see Fig. 3a and Supplementary Fig. S10 for examples of dissociation curves of five samples and Supplementary Figs. S11 and S12 for representative dissociation curves for each of the 12 samples). After 1200 µm, this trend was no longer consistent. It is at approximately this point that the competing phenomenon, which causes general loss in signal intensity, seems to play an increasingly important role (Supplementary Fig. S8). Since this decay overlaps with the effect that we want to monitor, we chose to avoid this complicating effect by analyzing the range of explosion widths that still have high signal intensity but where the decay phenomenon plays a smaller role, namely the region of interest 620–1200 µm (Fig. 3 and Supplementary Figs. S11 and S12).

In this range, DNA samples with lower theoretical T_m_ values showed more dissociation than DNA with higher binding affinities, as expected. In order to minimize the influence of individual data points on the results, data in the region of interest were fitted linearly. This fit was used to interpolate the relative amount of ssDNA (% ssDNA) at 1.1 mm explosion width, a point at which spectral signal-to-noise should be near maximum. This interpolated point was taken as the measure of dsDNA binding strength obtained from the LILBID laser dissociation curves. The % ssDNA at 1.1 mm strongly correlated with both solution state T_m_s and K_d_s, indicating that LILBID-MS laser dissociation curves reliably reflect dsDNA binding affinities (Fig. 3b). The dsDNAs designed for the three different 35mer sequences represent different lengths of dsDNA, a wide range of binding affinities, as well as different GC contents. Solution state binding affinity was the primary predictor of laser dissociation curve results independently of GC content or of 35mer used (Fig. 3b).

### Evaluating the reproducibility of the LILBID laser dissociation curve method

Results from LILBID laser dissociation curves collected on 16 different days were plotted against melting temperature and against log K_d_s (Supplementary Fig. S13). The minimal variation among these data demonstrates the reproducibility of this method. For all dsDNA samples, the variation between results from different days was similar to or less than the error propagated from the linear fit from one day’s results. Relatively little dissociation was observed for the most stable sample, the 20mer + 35mer dsDNA, indicating that the sample may approach the upper limit of binding affinity where laser-induced dissociation can still be achieved with the current LILBID ion source. Thus, this sample may indicate the maximum binding strength presently assessable with this method.

### Creating models for predicting binding affinities from LILBID laser dissociation curves and calculating the binding affinity of three DNA sequences using LILBID laser dissociation curves

**Figure 4:**
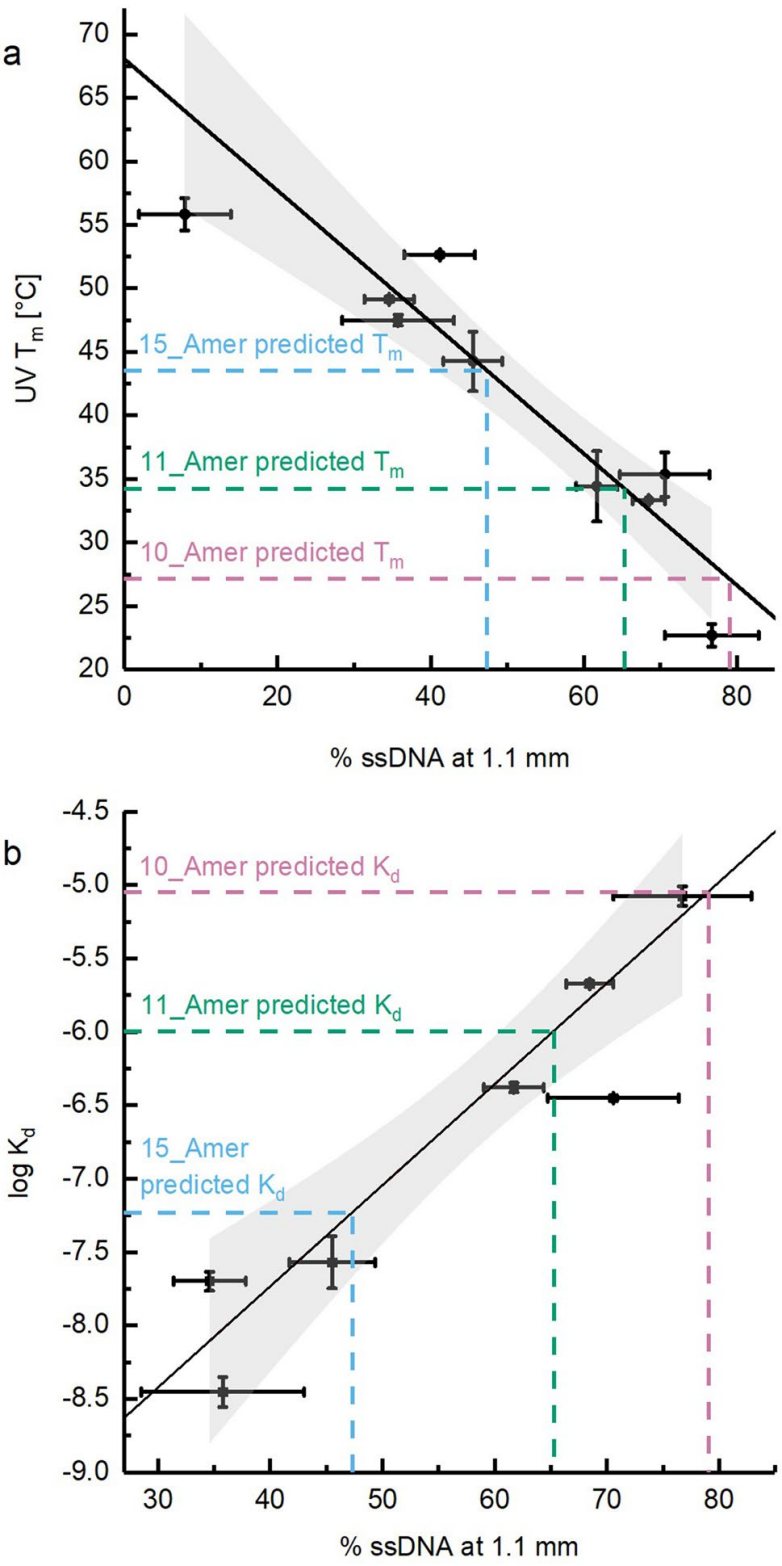
Linear models for predicting solution state binding affinities using the LILBID dissociation curve method and calculation of the solution state binding affinity of three dsDNA samples. Solution state binding affinities are plotted (**a**: T_m_, **b**: log K_d_) vs. % ssDNA at 1.1 mm (filled squares), shown as mean ± s.d. These points were fit with a linear fit weighted with the errors (s.d.) in x and y, using the York method^35^ and assuming no correlation between errors in x and in y. These fits yielded the models shown here for predicting T_m_ and log K_d_ from LILBID dissociation curves. The light grey areas represent the 95% confidence intervals yielded from these fits. Also shown are the % ssDNA for the three test samples (10_Amer, 11_Amer and 15_Amer dsDNAs) and the solution state binding affinities predicted for them from the % ssDNA using the linear models. This figure was prepared in OriginPro 2017 (https://www.originlab.com/).

We then asked whether the correlation between LILBID laser dissociation curve results and solution state results could be employed to establish a model for predicting DNA binding affinities. Of the 12 dsDNA samples, nine were selected to create models correlating T_m_ and K_d_ results with LILBID laser dissociation curve results. These nine samples were selected to include the samples with the highest and lowest T_m_s and cover a wide range of GC content. They include three with the 35_Amer as a scaffold, three with the 35_Bmer and three with the 35_Cmer. The remaining three samples will be used as test cases. For the nine selected dsDNAs the plot of melting temperatures versus % ssDNA at 1.1 mm was fitted to obtain a model correlating T_m_ and the % ssDNA obtained at 1.1 mm from LILBID laser dissociation curves (Fig. 4a). If this method is robust for different DNA sequences, then it should allow the prediction of a T_m_ for a sample based on the % ssDNA at 1.1 mm from LILBID:

1 $${\text{Predicted}}\;{\text{T}}_{{\text{m}}} = - 0.{52}\;^{ \circ } {\text{C }}\left( {\% \;{\text{ssDNA}}\;{\text{at}}\;{1}.{1}\;{\text{mm}}} \right) + {68}^{ \circ } {\text{C}}$$

Of the nine samples in the model set, two could not be evaluated with ITC because they fell outside of the range accessible with the method. ITC results for the remaining seven dsDNA samples were used to calculate an analogous empirical model for predicting K_d_ values from LILBID dissociation curve results (Fig. 4b):

2 $${\text{Predicted}}\;{\text{log K}}_{{\text{d}}} = 0.0{69 }\left( {\% \;{\text{ssDNA}}\;{\text{at}}\;{1}.{1}\;{\text{mm}}} \right) - {1}0.{5}$$

In order to test the accuracy of the LILBID laser dissociation method, these models were used to predict T_m_s and K_d_s for the remaining three samples (10_Amer, 11_Amer and 15_Amer dsDNAs) from the LILBID laser dissociation curve results (Fig. 4). For each of the three samples, five dissociation curves were obtained. T_m_s predicted from the fifteen individual LILBID curves fell within 3 °C of the UV measured values in 9/15 instances, within 5 °C in 12/15 instances and within 10 °C in 14/15 instances. Log K_d_s predicted from individual LILBID curves fell within 0.5 of ITC log K_d_s in 6/15 instances and within 1 in 14/15 instances.

Since typical protocols for ITC and UV measurements involve running multiple replicates in order to obtain sufficient statistics, we also considered the binding affinities predicted from the mean results of the five replicate LILBID curves per sample as shown in Table 3. The T_m_s predicted from means of five replicate LILBID curves differ minimally from the UV T_m_s and reflect the binding affinities of the samples, with the best agreement for the 10_Amer and 11_Amer samples. The log K_d_s predicted from five replicate LILBID measurements are within 0.50 of the measured values for all three samples.

**Table 3:** Measured and predicted T_m_s and log K_d_s. Each value represents the mean of all replicates.

Sample	T_m_ [°C]			log K_d_		
	Measured with UV	Predicted from LILBID	Predicted from ITC	Measured with ITC	Predicted from LILBID	Predicted from UV
10_Amer	26.0	27.1	31.2	− 5.42	− 5.05	− 4.70
11_Amer	34.6	34.3	39.1	− 6.45	− 6.00	− 5.89
15_Amer	40.0	43.5	46.1	− 7.51	− 7.23	− 6.65

*Note* nine dsDNAs were measured with UV and with LILBID, but only seven of these could be measured with ITC. Thus, models involving ITC measurements include seven dsDNAs, whereas all other models include nine dsDNAs.

To assess the quality of these results we wanted to compare our LILBID based predictions with those coming from the two other established methods we used here. An analogous linear model for predicting ITC results from T_m_s from individual UV curves showed a similar level of success in predicting log K_d_s: they fell within 0.5 in 1/8 instances and within 1 in 7/8 instances (Supplementary Fig. S14).

## Discussion

The most established solution state method and the primary MS-based method for assessing DNA binding affinities are UV melting curves^18,36–38^ and nESI melting curves^25^, respectively. Thus, the intuitive choice for assessing DNA binding with LILBID was to use a heatable droplet generator to create LILBID melting curves. While LILBID melting curve experiments yielded usable melting temperatures, the approach had severe limitations: LILBID melting curves could not access melting temperatures below 40 °C or above 55 °C (Supplementary Fig. S1). We therefore changed approaches and used variation of the LILBID laser to cause dissociation, introducing the LILBID laser dissociation curve method as a strong alternative to MS-based melting curve methods. LILBID laser dissociation curves access a wide range of binding affinities (T_m_s from the low 20 °C to the mid 50 °C range). In addition to having a wider range, the LILBID laser dissociation curve method has several inherent advantages. Each droplet yields one data point in a LILBID laser dissociation curve. For MS based melting curves (LILBID or nESI), spectra must be averaged over a period of time for each temperature point. Additionally, for LILBID or nESI melting curves the sample must be equilibrated at each temperature; during this time sample is lost out of the capillary. Unlike melting curves, LILBID laser dissociation curves require no temperature equilibration, thus involving much lower sample consumption and shorter measurement times compared to melting curves. While a good LILBID melting curve requires the measurement of 1000 droplets per temperature setting for a total of about 20,000 droplets (4 µl), for a LILBID laser energy dissociation curve about 300 droplets are measured (50 nl). Data for a LILBID melting curve can be collected in 2–4 h. In contrast, a full LILBID laser dissociation curve can be run in about 10 min.

Further, the LILBID laser dissociation curve method presents several advantages over the established solution state methods used in this study: UV melting curves and isothermal titration calorimetry. For a typical LILBID laser dissociation curve, normal handling procedures require 2 µL of 10 µM sample (20 pmol of each strand) to be loaded and measured within 10 min. For a reliable binding affinity, we suggest collecting three curves, for a total instrument time of 30 min. In contrast, the UV method required 200 µL of 10 µM sample (2 nmol of each strand) over a period of three hours to obtain two melting curves, and ITC required 300 µL of at least 2.5 µM 35mer, 40 µL of at least 30 µM complementary strand, and 1.5 h for each of three titrations (total: 2.3 nmol 35mer, 3.6 nmol complementary strand, 4.5 h). The LILBID method was able to quantify binding affinities for dsDNA with K_d_s in the range of 9 µM to 4 nM as well as for two dsDNA samples (the 12_Cmer and 20_Amer dsDNAs) with a binding affinity stronger than those measurable with standard ITC^20^. The upper limit of binding affinities accessible with our method is likely represented by the 20_Amer, since dsDNA with higher binding affinities than the 20_Amer would likely show no laser-induced dissociation. An analogous lower limit should exist where the sample is fully dissociated at all droplet explosion widths. However, with current mass resolution and peak broadening due to the 0.5 mM MgHPO_4_ buffer, the limiting factor is spectral resolution. With oligonucleotides smaller than a 8mer or 9mer, resolving the 35mer^-^ and dsDNA^-^ peaks becomes increasingly difficult. This will be overcome with future LILBID instruments.

In this first study on LILBID laser dissociation curves, it was also important to check the reproducibility of the new method. The image-based strategy used for the laser dissociation curves addresses many factors involved in laser-based dissociation at once. Small differences in droplet position relative to the LILBID laser beam are unavoidable from droplet to droplet. This affects the overlap of the desorption laser with the droplet and thus the amount of laser energy transferred to the sample, causing changes in both signal-to-noise and the amount of laser-induced dissociation. With the use of droplet explosion width as a measure of transferred laser energy, changes due to variations in droplet position are implicitly taken into account in LILBID laser dissociation curves. Any fluctuation in laser power from day to day would also be accounted for. The method is thus inherently robust against small changes in the setup, as indicated by the small variation in data collected on different days.

Double-stranded DNA oligonucleotides presented themselves as ideal samples for this proof-of-principle study, given the convenience of designing a series of similar dsDNAs with a large range of binding affinities. As our next step, we plan to extend the LILBID laser dissociation curve method to noncovalent protein–ligand and protein–protein complexes. MS-based binding affinity experiments with protein complexes typically involve titrations, as melting curve experiments require samples to be stable at high temperatures and are often not suitable for proteins. In contrast to titration-based experiments, LILBID laser dissociation curves can be obtained from pre-formed complexes and do not require separation of each component of a complex in advance. It is also not necessary to prepare and measure a series of samples for LILBID laser dissociation curves; one sample is sufficient. This new LILBID method will also save on sample consumption and measurement time for protein complexes. As demonstrated in this study, initial results with protein complexes could be verified by comparison with ITC results.

## Conclusion

In this study, we compare dsDNA binding affinities obtained with a new LILBID-based laser dissociation method to those from the standard UV melting curve and ITC experiments observed in the solution state. LILBID laser energy dissociation curves yielded binding affinity information over a large range (K_d_s from low µM to low nM). Strong correlation between binding affinities observed with the LILBID laser dissociation curves and solution state binding affinities (both melting temperatures and dissociation constants) together with the prediction of the binding affinities of three dsDNA samples demonstrate that LILBID laser dissociation curves can reliably yield quantitative binding affinities. With strong correlation to solution phase data, inherent robustness against sources of error, and practical advantages over melting curve- and titration-based studies, the LILBID laser dissociation curve method presents a promising alternative to existing methods and shows potential as a new way to quantitatively assess the binding affinities of noncovalent biomolecular complexes.

## Methods

### Sequence design

Twelve DNA sequences were designed to each bind one of three 35 bp long DNA sequences (scaffolds) for a total of 12 different dsDNA samples with 12 different theoretical T_m_s. The 35mer scaffolds were named 35_Amer, 35_Bmer and 35_Cmer, respectively, and have different GC contents. The 12 shorter sequences were named by their length as well as their corresponding scaffold: length_scaffoldmer (Table 1). The difference in length between the two single strands (ss) in each dsDNA sample (the scaffold and the shorter DNA sequence) minimized peak overlap in the LILBID-MS spectra, which would have hampered data analysis. Each of the shorter oligonucleotide sequences was designed to bind to only one location on the corresponding 35mer. Self-complementarity and hairpin formation were also avoided in all dsDNA samples by checking their likelihood with the online OligoAnalyzer Tool from Integrated DNA Technologies (Coralville, USA) (https://eu.idtdna.com/calc/analyzer). The set of dsDNA samples was designed to cover a range of binding affinities as shown in Table 1. In order to confirm that any results observed were not caused by sequence length alone, sequences with the same length but different GC content and thus different binding affinities were included (Table 1).

### Sample preparation

DNA oligonucleotides were purchased as desalted, dry custom oligonucleotides from Life Technologies (Darmstadt, Germany) and stored at a concentration of about 200 µM in water at 4 °C. A stock solution of 1 mM MgHPO_4_ (Alfa Aesar, Haverhill, Massachusetts / USA) was adjusted to pH 7.2 using dilute HCl. The dsDNA samples were prepared by combining Millipore water and ssDNA and MgHPO_4_ stock solutions to reach the final concentrations of 10 µM of each ssDNA and 0.5 mM MgHPO_4_. This buffer was chosen because its pH remains relatively stable over our temperature range, and it demonstrated sufficient buffer capacity for this sample concentration. dsDNA samples were annealed by heating at 95 °C for 10 min and cooling to room temperature overnight.

### UV absorption melting curves

UV melting curves were recorded with a Jasco J-810 spectropolarimeter with a Peltier temperature controller. For each 200 µl sample, a UV absorption spectrum was recorded at a path length of 1 mm. A UV melting curve was then recorded at the wavelength of maximum absorption. The melting point of each curve was determined by finding the maximum of the first derivative.

### Isothermal titration calorimetry

ssDNA solutions were prepared by combining Millipore water and ssDNA and MgHPO_4_ stock solutions to reach the desired DNA concentrations and 0.5 mM MgHPO_4_. ITC experiments were performed at 25 °C with an iTC200 microcalorimeter (Malvern Panalytical, Malvern, UK) with the 35mer ssDNA in the sample cell and the complementary strand in the syringe. Data were analysed using the software provided by MicroCal in Origin 7 (Originlab, Northampton, MA, US) assuming one-site binding. At least three titration curves were collected per sample.

### LILBID mass spectrometry

LILBID-MS spectra were collected using a home-built mass spectrometer with a LILBID ion source and ToF detector, described in more detail by Morgner et al.^10^. In this setup, droplets of aqueous sample are emitted at 10 Hz from a piezo-driven droplet generator (Microdrop Technologies, Norderstedt, Germany). The droplets emerge from a 50 µm diameter glass capillary into a 100 mbar environment and are transferred into vacuum (10^–5^ mbar). Each droplet is irradiated by a ~ 6 ns laser pulse at 3 µm, causing it to undergo an explosive expansion, releasing sample ions. After a delay time of 6 µs, ions are accelerated out of the ion source and mass-analysed by time-of-flight. LILBID settings were chosen to not bias the relative response factors of dsDNA^-^ and 35mer^-^ peaks (see Supplementary Tables S1 and S2).

Percent ssDNA was calculated as the 35mer^-^ peak area divided by the sum of dsDNA^-^ and 35mer^-^ peak areas and multiplied by 100. The peaks corresponding to the shorter oligonucleotides were not used for the analysis either due to spectral overlap or because they were outside of the optimal m/z range for these LILBID settings. Dissociation was observed at all charge states; however, due to peak overlap at higher charge states for some samples, only the singly charged state was used for further analysis. The 10_Amer + 35_Amer sample was chosen to verify that this simplification does not introduce bias, as the peak overlap for the second charge state is minimal in this sample. The data analysis for this sample was repeated with the second charge state included in the calculation of % ssDNA: 100*(35_Amer^-^ + 35_Amer^2-^)/(35_Amer^-^ + 35_Amer^2-^ + dsDNA^-^ + dsDNA^2-^). The resulting 10_Amer dissociation curve gives the same results as that calculated from the first charge state alone, confirming the validity of calculations using only the first charge state (Supplementary Fig. S15). At these LILBID settings, the relative response factors for DNA are very similar within the m/z range covered by the dsDNA^-^ and 35mer^-^ peaks (Supplementary Table S2).

### LILBID laser dissociation curves

**Figure 5:**
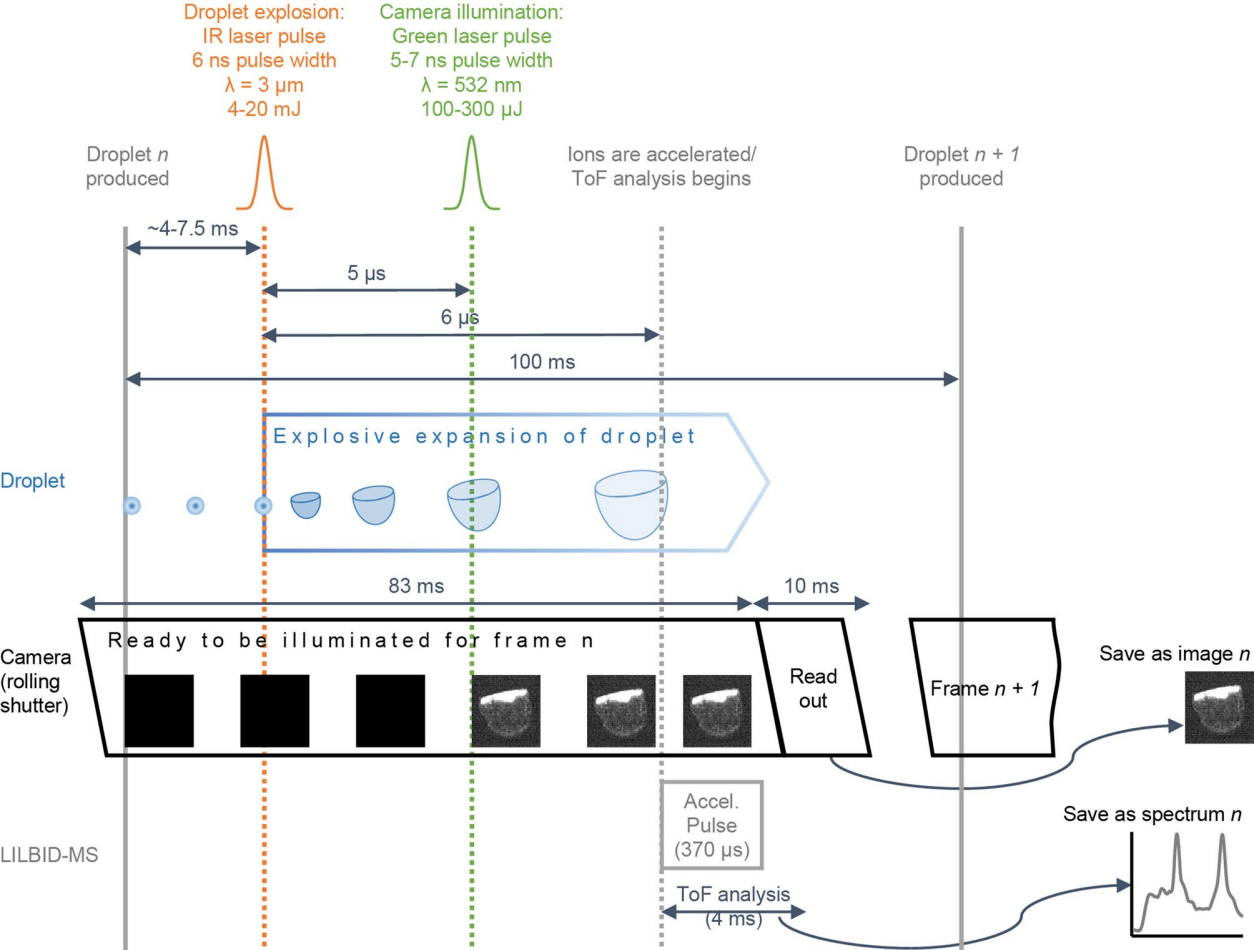
Timing and settings of lasers, droplet emitter, camera, LILBID-MS, and data readout for LILBID laser dissociation curves. The infrared laser pulse illuminates the droplet, resulting in its explosive expansion. Five µs after the beginning of the droplet explosion, the droplet is illuminated with a pulsed green laser, which allows the capture of the droplet explosion on the camera. One camera frame is produced per droplet. For each frame, the camera’s exposure time is 83 ms, and light arriving at the camera detector would be added over the entire exposure time. However, since the region of interest is only illuminated during the green laser pulse (5–7 ns pulse width), a snapshot of the droplet is recorded. One µs after droplet illumination, ions are accelerated into the ToF, and ToF analysis begins. The camera readout and ToF readout for each droplet *n* are saved as image *n* and spectrum *n*, respectively. The exposure time for frame *n* + *1* begins before the emission of the droplet *n* + *1.* Laser energy was measured outside the ion source. This diagram was prepared in Powerpoint 2013 (https://www.microsoft.com/en/microsoft-365/previous-versions/microsoft-powerpoint-2013). The images contained in this diagram were prepared using Fiji^33^.

To achieve laser energy dependent dissociation curves in LILBID, samples were measured at room temperature. Droplets were emitted at 10 Hz, and droplet position at the time of laser pulse was continuously varied by adjusting the time between droplet production and laser irradiation. This resulted in different amounts of overlap between the droplet surface and the laser beam and different amounts of laser energy being transferred to the sample. Each resulting droplet explosion was illuminated 5 µs after the IR laser pulse using a Minilite I laser (Continuum, San Jose, USA) (pulse length: 5–7 ns pulse length at FWHM) and imaged with a DFK 23UP031 camera (Imaging Source, Bremen, Germany) at a resolution of 15 µm per pixel. One mass spectrum was also recorded per droplet and numbered so that each spectrum could be matched to the corresponding droplet explosion image. Figure 5 gives an overview of the settings and timings of the droplet generator, lasers, camera, LILBID-MS and data readout.

Explosion images were analysed in Fiji^33^: each image was 2 × 2 binned to reduce noise and converted to black and white by setting a manual threshold. The width of the explosion was measured by calculating the Feret’s diameter of the top part of the explosion; the error in this measurement stems primarily from the binned pixel size (pixel = 31 µm). Mass spectra were calibrated using a two point calibration, smoothed using a moving average over a range of 20 m/z, and linearized in Python. Peak areas were calculated in OriginPro 2017. The background for each peak was defined by setting a line between the minimum in the spectrum to the left of the peak and the minimum to the right of the peak. Peak area was calculated by integrating over this range and subtracting the area under the background line. For spectra where the 35mer^-^ and dsDNA^-^ peaks were not baseline resolved, the 35mer^-^ peak area was calculated by integrating background subtracted spectra from the minimum to the left of the peak to the minimum between the two peaks. The dsDNA^-^ peak area was set as the area integrated from the minimum between the two peaks to the minimum to the right of the peak. Dissociation curves were plotted as % ssDNA vs. explosion width.

## Supplementary information

Supplementary Information.

## Data Availability

The datasets generated and analyzed in this paper, together with the Python and Fiji scripts used for data interpretation can be accessed at Zenodo (https://doi.org/10.5281/zenodo.4088566).
