# Supplementary material for: LILBID laser dissociation curves: a mass spectrometry-based method for the quantitative assessment of dsDNA binding affinities
Source: Sci Rep. 2020 Nov 23;10:20398. doi: 10.1038/s41598-020-76867-9 (PMC7683618; doi:10.1038/s41598-020-76867-9)
Supplement: Supplementary file 1 — Supplementary Information. [file 41598_2020_76867_MOESM1_ESM.docx]

*Supporting Information*

LILBID-MS based method for the quantitative assessment
of dsDNA binding affinities.

Phoebe Young^1^, Genia Hense^1^, Carina Immer^2,3^, Jens Wöhnert^2,3^, Nina Morgner^1^.

^1^Institute of Physical and Theoretical Chemistry, J.W. Goethe University, Frankfurt am Main, Germany.

^2^Institute for Molecular Biosciences, J.W. Goethe University, Frankfurt am Main, Germany.

^3^Center for Biomolecular Magnetic Resonance (BMRZ), J.W. Goethe University, Frankfurt am Main, Germany.

**Table of Contents**

Supplementary Table S1.…….……………………………..…...…………………..…………………S-2

Supplementary Table S2…………………….…….…………..……………………..…………………S-3

Supplementary Figure S1………………….…………………………………………...………………S-4

Supplementary Figure S2..…………………..…………………………………………………………S-5

Supplementary Figure S3…..………………………………..…………………………………………S-6

Supplementary Figure S4…..…………………………..………………………………………………S-7

Supplementary Figure S5………………………………………..………………..……………………S-8

Supplementary Figure S6…..………………………………………………..…………………………S-9

Supplementary Figure S7…..………………………………………………..………………………..S-10

Supplementary Figure S8…..………………………………..……………..…………………………S-11

Supplementary Figure S9…..……………………………………..………..…………………………S-12

Supplementary Figure S10..………………………………………………..…………………………S-13

Supplementary Figure S11..………………………………………………..…………………………S-14

Supplementary Figure S12..………………………………………………..…………………………S-15

Supplementary Figure S13..………………………………………………..…………………………S-16

Supplementary Figure S14..………………………………………………..…………………………S-17

Supplementary Figure S15..………………………………………………..…………………………S-18

Supplementary Table S1: Relative LILBID response factors for DNA in the m/z range between 10.8 kDa and 17.1 kDa. Samples were prepared with four different ratios of the 35_Amer ssDNA and a 55mer ssDNA in 0.5 mM MgHPO_4_, pH 7.5 and measured with LILBID-MS using the data collection and analysis methods for the LILBID laser dissociation curves. The 55mer is a ssDNA with a molecular weight similar to the largest dsDNA (20_Amer + 35_Amer) used in this study (5’ to 3’ sequence: TTG TAG TTT TTT TGA GTT GAA TTG TAG TTT TTT TGA GTT GAT TAT GTT TTT AGT A). The 55mer does not bind the 35_Amer. The % 35_Amer (100*35_Amer^-^/(35_Amer^-^ + 55mer^-^)) calculated at 1.1 mm explosion width differs minimally from the theoretical % 35mer. This indicates that at the given settings, the LILBID dissociation method would introduce only minimal bias through differences in the relative response factors of the 35mers and various dsDNAs analyzed in this paper.

| **sample** | **Conc. of 35_Amer [µM]** | **Conc. of 55mer [µM]** | **Theor. % 35_Amer** | **Exp. % 35_Amer at 1.1 mm** |
| --- | --- | --- | --- | --- |
| 1 | 10 | 5 | 67 | 69 |
| 2 | 10 | 10 | 50 | 55 |
| 3 | 5 | 10 | 33 | 40 |
| 4 | 5 | 5 | 50 | 56 |

Supplementary Table S2: Influence of delayed extraction on the percent ssDNA calculated at 1.1 mm explosion width. Delayed extraction was defined as the time between laser desorption and acceleration of the ions into the ToF detector. A mixture of 10 µM 35_Amer and 10 µM 55mer ssDNAs in 0.5 mM MgHPO_4_, pH 7.5 was run on LILBID-MS using different delayed extraction times. The data collection and analysis methods for the LILBID laser dissociation curves were used. The 55mer is a ssDNA with a molecular weight similar to the largest dsDNA (20_Amer + 35_Amer) used in this study (5’ to 3’ sequence: TTG TAG TTT TTT TGA GTT GAA TTG TAG TTT TTT TGA GTT GAT TAT GTT TTT AGT A). The 55mer does not bind the 35_Amer. The % 35_Amer (100*35_Amer^-^/(35_Amer^-^ + 55mer^-^)) calculated at 1.1 mm explosion width does not vary significantly in this range of delayed extraction times. This indicates that at the given settings, the delayed extraction does not bias the results of LILBID-MS laser dissociation studies. All other LILBID-MS experiments in this study were carried out with 6 µs delayed extraction. Three sets of 100 spectra and images were collected per delayed extraction setting.

| **Delay [µs]** | **Theoretical % 35_Amer** | **% 35_Amer at 1.1 mm** |
| --- | --- | --- |
| 2 | 50 | 55 |
| 4 | 50 | 54.6 |
| 6 | 50 | 55 |
| 8 | 50 | 57.6 |
| 10 | 50 | 53.0 |

Supplementary Figure S1: UV- and LILBID-based melting curves for four dsDNA samples. LILBID melting curves were run using a droplet generator with a sample heating option (Microdrop, Norderstedt, Germany), starting at room temperature, increasing the temperature in 2°C increments, and ending at the highest temperature at which the droplet production was still consistent enough to permit uniform transfer of droplets into the ion source. At each temperature, the sample was allowed to equilibrate for 2 min before at least two sets of 500 droplets were analysed. Each set of 500 spectra was averaged before data analysis. These averaged spectra were calibrated, smoothed, and linearized in Massign [39] before background correction and peak integration in OriginPro 2017 (Originlab, Northampton, MA, US). Melting curves were created by plotting the mean % ssDNA for the sets of spectra measured at each temperature against temperature. The melting temperatures were calculated where applicable by fitting each melting curve using logistic regression. a) 10_Amer + 35_Amer, b) 15_Amer + 35_Amer, c) 15b_Amer + 35_Amer, d) 20_Amer + 35_Amer. UV-based melting curves (black) were baseline corrected as described by Owczarzy [34], except for the 10_Amer curve (a), where this was not possible. LILBID melting curves are shown in blue for a, c, and d; for the 15_Amer sample, two independent LILBID melting curves were performed (blue and green). Points represent mean % ssDNA from LILBID spectra collected at each temperature. T_m_s for each curve are shown as dotted lines in the corresponding colors. This figure shows that melting temperatures from LILBID and UV curves are comparable. Differences may stem from inherent differences between the two methods: for example, UV spectroscopy measures % dissociated base pairs, and LILBID measures the % fully dissociated DNA. For the uncorrected UV melting curves, see Supplementary Figs. S4.


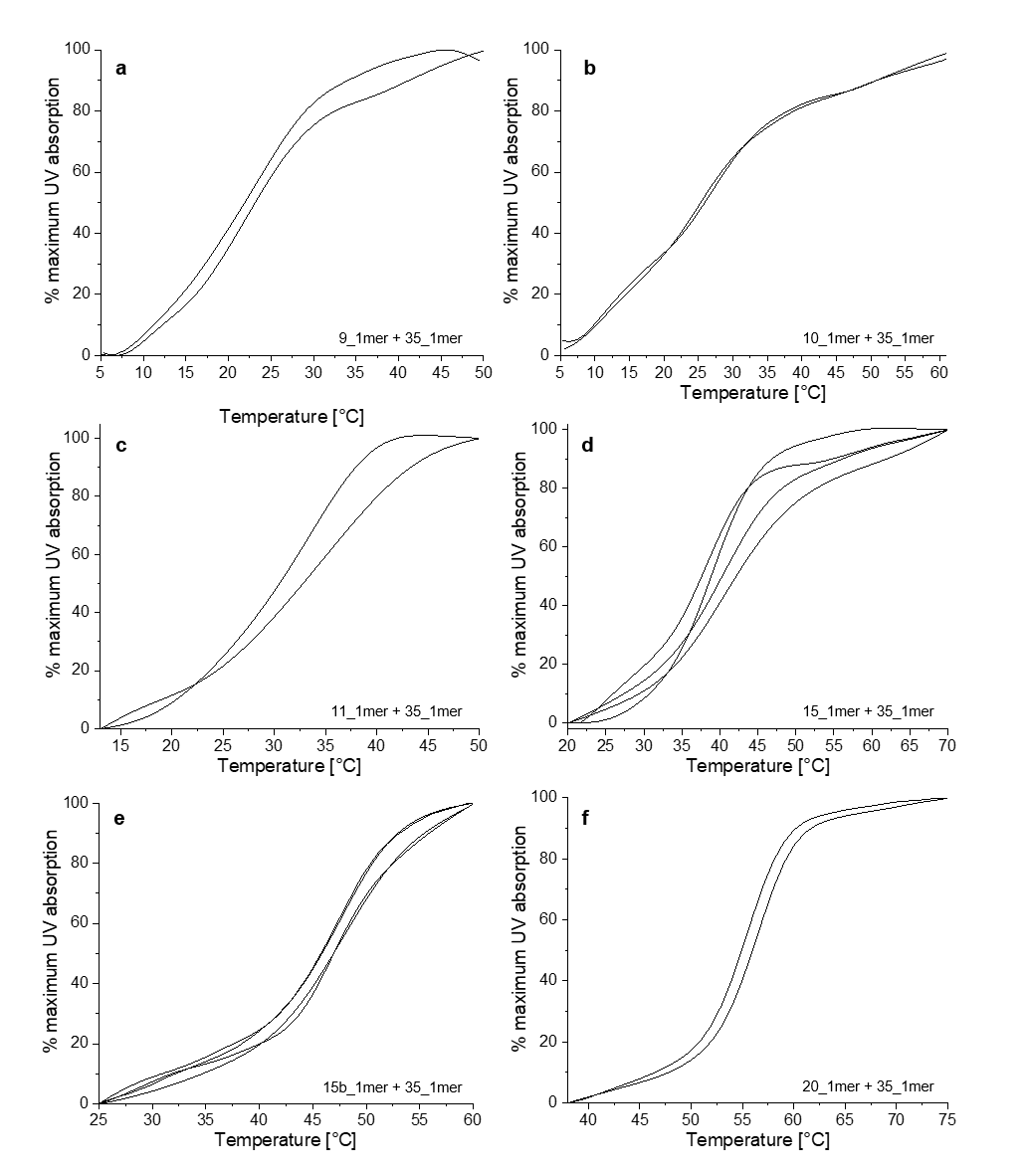
Supplementary Figure S2: Full UV absorption melting curves, plotted as % maximum UV absorption vs. temperature.


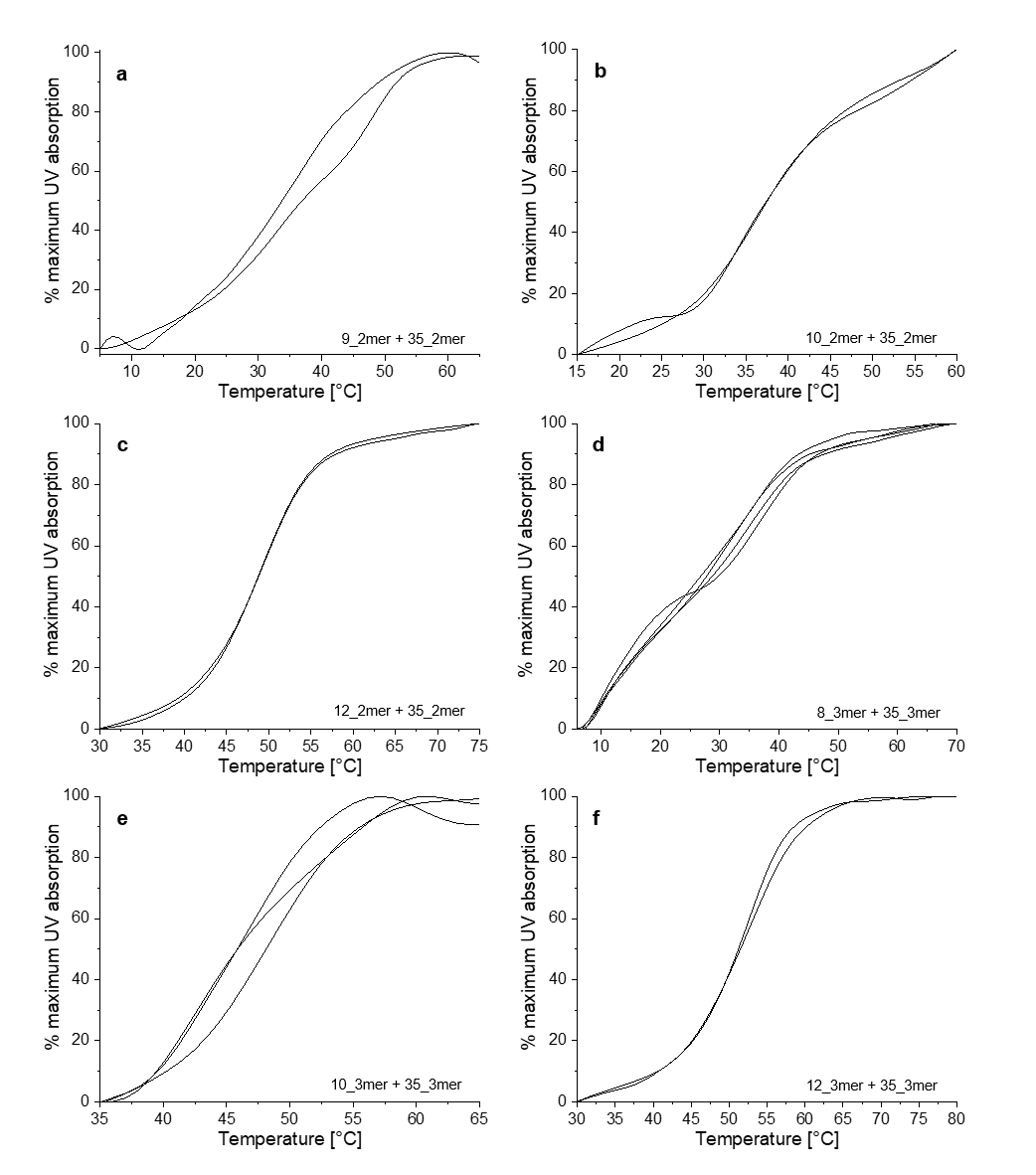
Supplementary Figure S3: Full UV absorption melting curves, plotted as % maximum UV absorption vs. temperature.


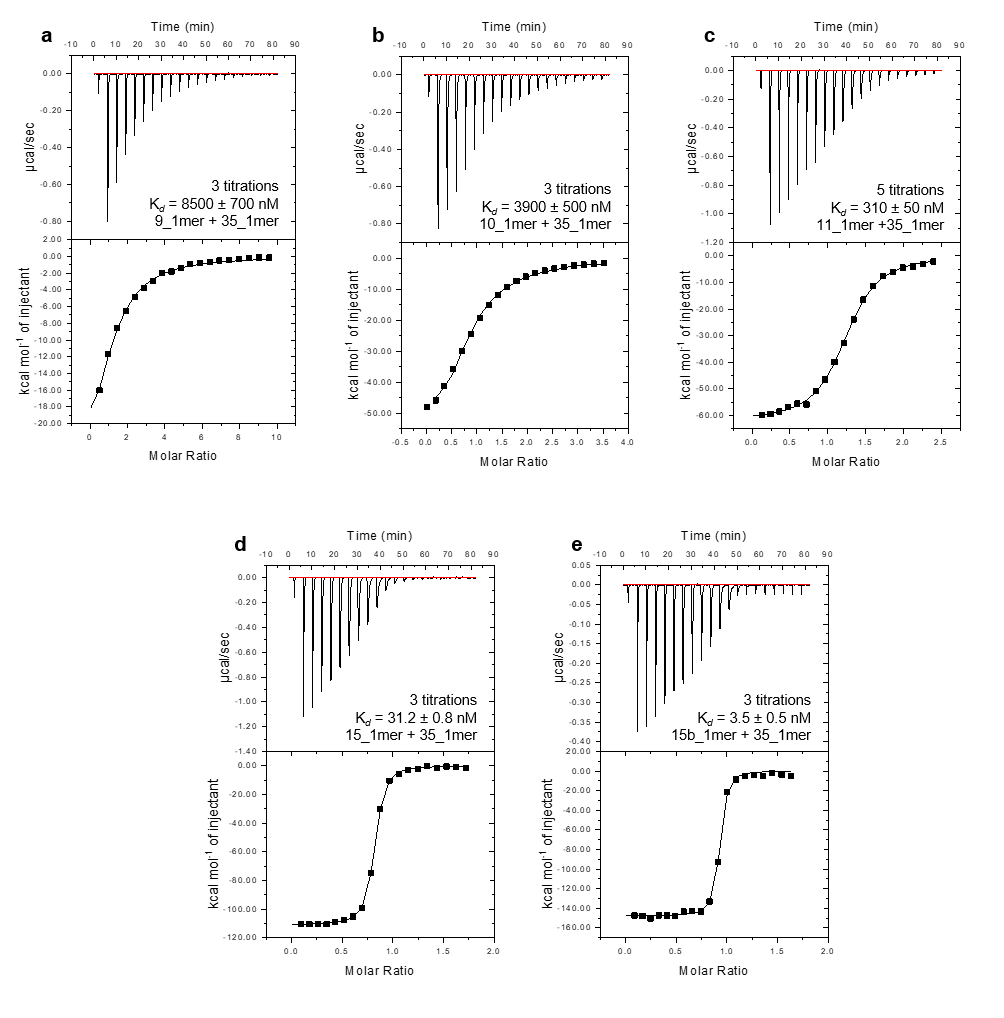


Supplementary Figure S4: ITC results for five dsDNA samples. Dissociation constants are given as mean ± s.e.m.


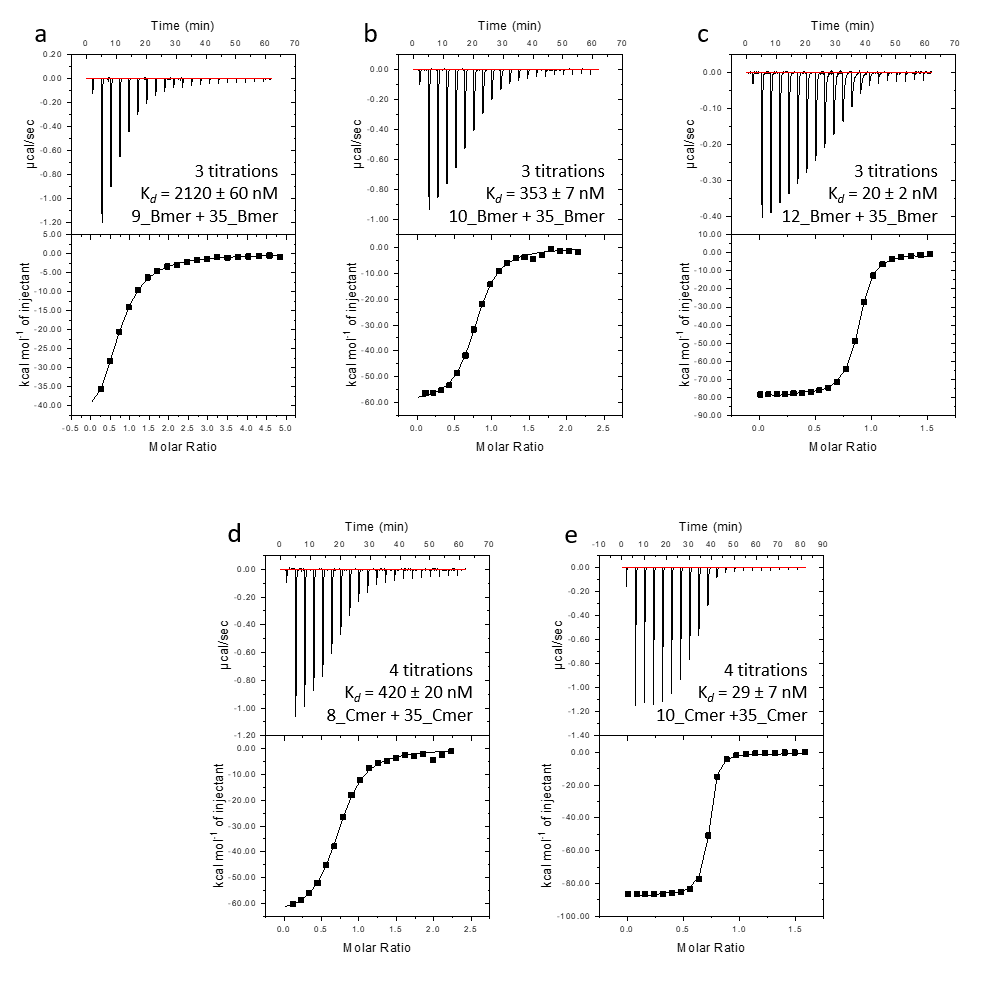


Supplementary Figure S5: ITC results for five dsDNA samples. Dissociation constants are given as mean ± s.e.m.

Supplementary Figure S6: Dependence of explosion size on buffer concentration at settings corresponding to two different levels of laser energy. The samples measured were 0, 0.5 and 1 mM 0.5 mM MgHPO_4_, pH 7.5 (■, ●, ▲, respectively). 0.5 mM MgHPO_4_, pH 7.5 corresponds to the buffer concentration used for all samples in this paper. For each sample, droplets were emitted and irradiated at 10 Hz while the user attempted to optimize laser energy transfer. Each cluster of five points represents the widths of the five largest explosions obtained from a set of 300 consecutive images; these should reflect the explosions obtained with optimum laser energy transfer at the given settings. For each set of five points, the mean value is represented with a short horizontal line. For each concentration and laser setting, four replicates of 300 images were recorded. The variations between replicates is greater than the variation between data from different test groups (buffer concentrations), indicating that unavoidable fluctuation in laser performance and thus laser energy transfer over the course of the measurements plays a greater role in explosion width than does buffer concentration.

Supplementary Figure S7: Dependence of explosion size on sample concentration at settings corresponding to two different levels of laser energy. The samples measured were 0 µM or 40 µM 15_Amer in 0.5 mM MgHPO_4_, pH 7.5 (black and white, respectively). 40 µM 15_Amer corresponds to the highest concentration of DNA used in this paper (20 µM dsDNA). For each sample, droplets were emitted and irradiated at 10 Hz while the user attempted to optimize laser energy transfer. Each cluster of five points represents the widths of the five largest explosions obtained from a set of 300 consecutive images; these should reflect the explosions obtained with optimum laser energy transfer at the given settings. For each set of five points, the mean value is represented with a short horizontal line. For each concentration and laser setting, four replicates of 300 images were recorded. The variations between replicates is greater than the variation between test groups (sample concentrations), indicating that unavoidable fluctuation in laser performance and thus laser energy transfer over the course of the measurements plays a greater role in explosion width than does sample concentration. The variations between measurements of the same samples in Fig S-6 and S-7 also underscore the need for a method that is tolerant of differences in laser energy transfer.

Supplementary Figure S8: Hypothetical model for the initial increase and later decrease in signal intensity observed for increasing explosion width. The range of explosion widths used for all further LILBID studies is also indicated: 620-1200 µm (region of interest).

Supplementary Figure S9: LILBID spectra of the 10_Amer + 35_Amer dsDNA sample corresponding to five droplets with different explosion widths. The spectra shown are those used in Fig. 2; here the full m/z range is depicted. Spectra were normalized to the peak height of the dsDNA^-^ peak. At the largest explosion widths, a small shoulder appears at the lower m/z side of the peaks. This reflects laser-induced loss of small fragments from the DNA strands at high levels of laser energy transfer. As this shoulder is included in peak integration, this fragmentation does not affect results.

Supplementary Figure S10: Unprocessed version of the LILBID laser dissociation curves shown in Fig. 3. Each point represents the % ssDNA and explosion width associated with the spectrum and image from one droplet. Data was used from all droplets where the spectra had significant signal-to-noise. In order to produce the curves shown in Fig. 3, the data points shown here were sorted by explosion width and grouped into groups of 5. A mean explosion width +/- s.e.m. and a mean % ssDNA +/- s.e.m. was calculated for each group. The resulting means, x error bars and y error bars were then plotted for Fig. 3. The linear fits shown in Fig. 3 were calculated via linear regression of all raw (ungrouped) points in the region of interest 620-1200 µm.


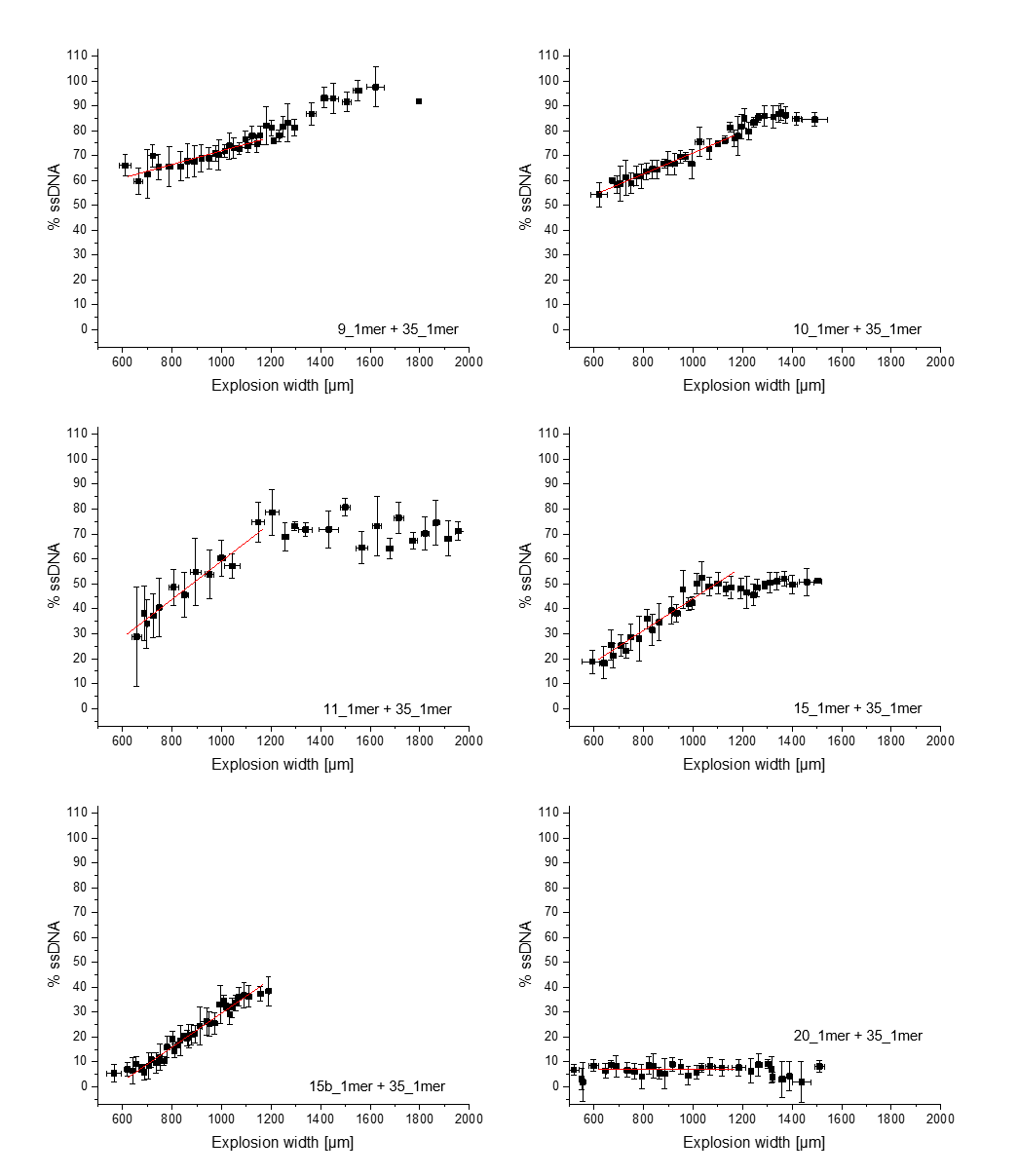
Supplementary Figure S11: LILBID laser energy dissociation curves for six dsDNA samples with linear fits in the region of interest (620 to 1200 µm).


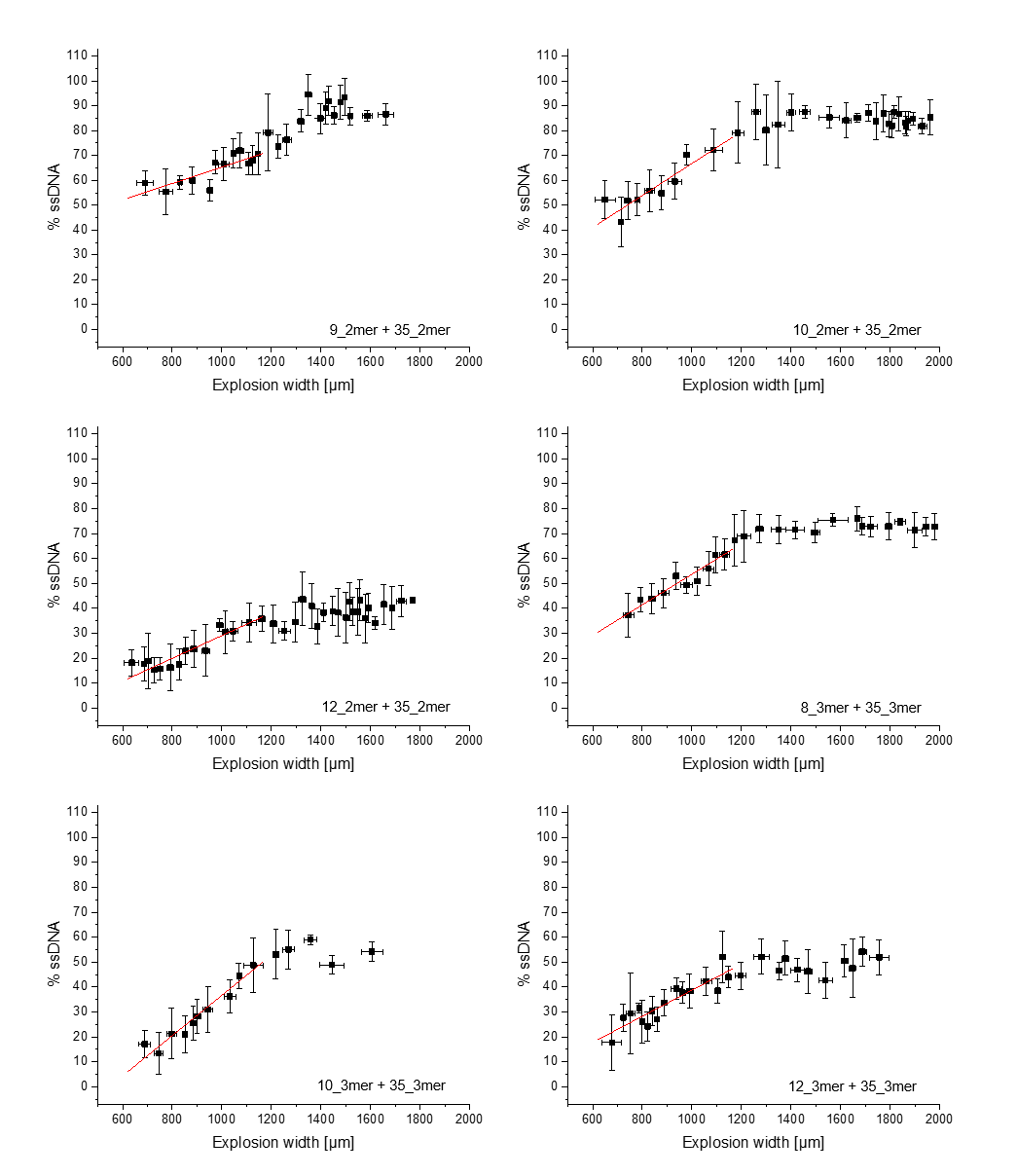


Supplementary Figure S12: LILBID laser energy dissociation curves for six dsDNA samples with linear fits in the region of interest (620 to 1200 µm).


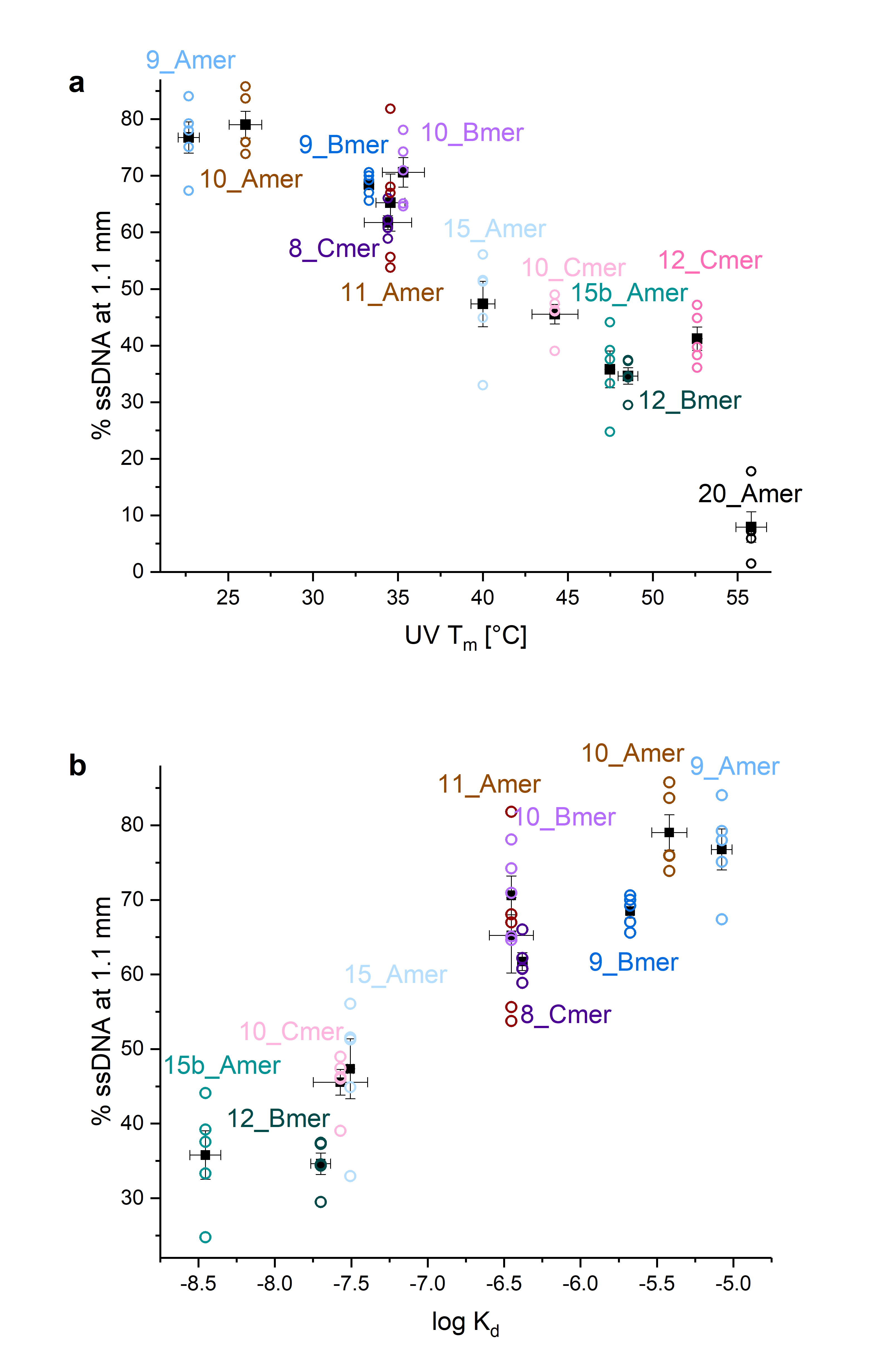


Supplementary Figure S13: Reproducibility of the LILBID dissociation curve method. Percent ssDNA at 1.1 mm vs. solution state binding affinity with five replicates per sample. Mean values for each sample are shown as black squares with the errors in y calculated as the s.e.m. a) % ssDNA vs. T_m_ for 12 dsDNA samples. T_m_ values on the x axis are mean values from UV melting curves ± s.e.m. b) % ssDNA vs. log K_d_ for 10 dsDNA samples. Log K_d_ values on the x axis are mean values from ITC replicates ± s.e.m.

Supplementary Figure S14: Prediction of log K_d_s for three dsDNA samples using the UV melting curves. Squares represent T_m_s vs. log K_d_s, shown as mean ± s.d. These points were fit with a linear fit weighted with the errors (s.d.) in x and y, using the York method [38] and assuming no correlation between errors in x and in y. This fits yielded the model shown here for predicting log K_d_ from UV melting curves. The light grey area represents the 95% confidence intervals yielded from the fit. Also shown are the T_m_s for the 3 test samples (10_Amer, 11_Amer and 15_Amer dsDNAs) and log K_d_s predicted for them from their UV T_m_s and using the linear model.

Supplementary Figure S15: 10_Amer + 35_Amer LILBID laser dissociation curves. % ssDNA peak area was calculated using one charge state or two charge states: 35_Amer^-^/(35_Amer^-^ + dsDNA^-^) (black) or (35_Amer^-^ + 35_Amer^2-^)/(35_Amer^-^ + 35_Amer^2-^ + dsDNA^-^ + dsDNA^2-^) (red).

**Additional References**

39 Morgner, N. & Robinson, C. V. Massign: an assignment strategy for maximizing information from the mass spectra of heterogeneous protein assemblies. Anal. Chem. 84, 2939-2948 (2012).
